# Case Report: Novel Compound-Heterozygous Variants of *SKIV2L* Gene that Cause Trichohepatoenteric Syndrome 2

**DOI:** 10.3389/fgene.2021.756451

**Published:** 2021-10-06

**Authors:** Qiao Zhang, Xia Qian, Jianli Zhou, Lin Han, Shaoming Zhou, Zhaoxia Wang

**Affiliations:** ^1^ Division of Gastroenterology, Shenzhen Children’s Hospital, Shenzhen, China; ^2^ Running Gene Inc., Beijing, China

**Keywords:** trichohepatoenteric syndrome (THES) 2, SKIV2L gene, intractable diarrhea, whole-exome sequencing (WES), pediatrics

## Abstract

**Background:** Trichohepatoenteric syndrome (THES) is a rare disease that mainly causes intractable diarrhea. It is classified into THES1 and THES2, which are associated with the tetratricopeptide repeat domain 37 (TTC37) gene and Ski2-like RNA helicase (SKIV2L) gene, respectively. THES is not very prevalent in China or worldwide, but new cases have increasingly been reported.

**Methods and Results:** Here, we report the clinical and genetic information of a 1.5-month-old girl who was admitted to our hospital due to diarrhea and failure to thrive. Whole-exome sequencing (WES) revealed novel compound-heterozygous variants of the SKIV2L gene, c.3602_3609delAGCGCCTG (p.Q1201Rfs*2), and c.1990A > G (p.T664A) as the causative factors, which were confirmed via Sanger sequencing. Upon continuous feeding with an amino-acid formula through a gastric tube and parenteral nutrition, the patient resumed thriving and her stool frequency decreased.

**Conclusion:** We report a girl carrying novel variants of the *SKIV2L* gene that cause THES2, thereby providing valuable information on the diagnosis of THES2 and expanding the spectrum of disease-causing *SKIV2L* mutations.

## Introduction

Trichohepatoenteric syndrome (THES), initially called syndromic diarrhea (SD), was first described by Stankler et al., in 1982 and coined by Verloes et al., in 1997 (Stankler et al., 1982; Verloes et al., 1997). The typical symptoms of THES are intrauterine growth retardation and neonatal intractable diarrhea, leading to poor weight gain and failure to thrive. Patients may also have hair abnormalities (trichorrhexis nodosa) or facial dysmorphisms (Verloes et al., 1997). Other clinical characteristics, including immunodeficiency, skin abnormalities, hepatic involvement, intellectual disability, and congenital heart disease, may also present. In Europe, the estimated prevalence of THES is 1:1,000,000, and the 10-years mortality rate is >50% (Fabre et al., 2013; Fabre et al., 2017). The mainstream management of THES is mainly based on parenteral nutrition and immunoglobulin supplementation.

THES is classified into THES1 (OMIM #222470) (69% of the cases) and THES2 (OMIM #614602) (31%). THES1 is caused by homozygous or compound-heterozygous mutations in the tetratricopeptide repeat domain 37 (*TTC37*) gene, whereas THES2 is associated with homozygous or compound-heterozygous mutations in the Ski2-like RNA helicase (*SKIV2L*) gene (Hartley et al., 2010; Fabre et al., 2012; Bourgeois et al., 2018). The clinical manifestations of these two types are identical. Two-thirds of reported THES cases were caused by pathogenic variants in *TTC37*, and the others were associated with mutations in the *SKIV2L* gene (Bourgeois et al., 2018). Both genes encode proteins that are components of the human super killer (Ski) complex, a cofactor of the RNA exosome, playing roles in the degradation of aberrant mRNAs (Schneider and Tollervey, 2013).

The *SKIV2L* gene (OMIM #600478) is located on chromosome 6p21.33 and has 28 exons. It encodes an RNA helicase (helicase SKI2W) of 137 kDa comprising 1,246 amino acids (Dangel et al., 1995; Lee et al., 1995; Yang et al., 1998) and contains a helicase superfamily ATP-binding domain that is involved in exosome-mediated RNA decay (Bourgeois et al., 2018). Previous reports have shown the associations of *SKIV2L* with several diseases, including age-related macular degeneration, inflammatory bowel disease, immunodeficiency (primary or common variable), and mitochondrial diseases (McKay et al., 2009; Kammermeier et al., 2014; van Schouwenburg et al., 2015; Stray-Pedersen et al., 2017; Riley et al., 2020). However, most mutations in *SKIV2L* have been reported to be responsible for THES2. To date, 45 *SKIV2L* mutations have been reported to the Human Gene Mutation Database (HGMD v2021.8) (Stenson et al., 2017), including 35 mutations associated with THES2. Of these THES2-causing mutations, only three were detected in the Chinese population (Zheng et al., 2016; Fung et al., 2020). Herein, we report a THES2 patient with two novel compound-heterozygous variants of the *SKIV2L* gene.

## Methods

### Clinical Examination

The medical history of this patient was provided by her parents and included the birth history, clinical manifestations, and diagnostic and therapeutic actions. Physical examination and laboratory tests were performed, including blood and fecal routine tests, fecal culture, and hepatic, renal, and immunological function tests. Imagological examinations, including abdominal ultrasound, cardiac Doppler ultrasonography, and brain magnetic resonance imaging (MRI), were also applied.

### Whole-Exome Sequencing

Whole-exome sequencing (WES) and Sanger sequencing were performed to identify the causative genes. Peripheral blood samples were collected from the patient and her parents and sent to Running Gene Inc. (Beijing, China). The whole sequencing process was described in a previous study (Chen et al., 2020). DNA samples were extracted from the blood samples, qualified, and fragmented into 200–300 bp fragments for library preparation. Probes were hybridized with the prepared libraries to capture exomes, according to the protocol of the Agilent Sure Select XT2 Target Enrichment System (Agilent, Santa Clara, CA). Captured DNA samples were sequenced on Novaseq 6,000 platform (Illumina, San Diego, CA). Raw data in FASTQ format were trimmed and filtered for quality control by using fastp (Chen et al., 2018). The qualified reads were aligned to human reference sequence GRCh37/hg19 by using BWA (Li and Durbin, 2009). Single-nucleotide variants (SNVs) and insertions/deletions (indels) were called out using GATK (Van der Auwera et al., 2013). All the called variants were annotated based on the genetic databases, including ExAC v1.0 (Lek et al., 2016), gnomAD v2.1.1 (Karczewski et al., 2020), ESP6500SI-V2 (Fu et al., 2013), 1kGenomes v3.7.6 (Auton et al., 2015), HGMD v2021.8 (Stenson et al., 2017), ClinVar (Landrum and Kattman, 2018), China National GeneBank (CNGB), and an in-house database (Access date: August 31, 2021). Variants with high allele frequency (>1%) were filtered out. The pathogenicity of variants was assessed based on the American College of Medical Genetics and Genomics (ACMG) guidelines (Richards et al., 2015). The *SKIV2L* mutations were finally selected based on its clinical relevance and pathogenicity.

## Results

### Case Presentation

**FIGURE 1:**
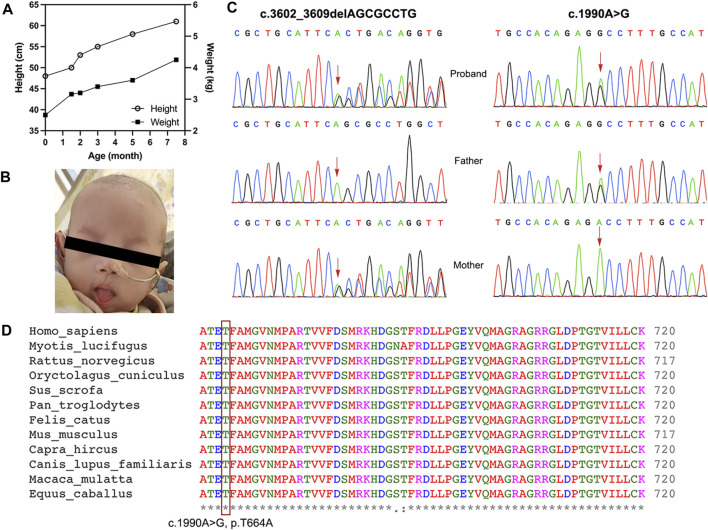
(**A**) The graphs show the height and weight of the patient during the treatment course. (**B**) The patient had woolly and brittle hair. (**C**) The compound-heterozygous *SKIV2L* mutations. The frameshift mutation with maternal origin, c.3602_3609delAGCGCCTG (p.Q1201Rfs*2), and missense mutation with paternal origin, c.1990A > G (p.T664A), were identified in the patient. (**D**) Threonine 664 is highly conserved across species. The level of conservation is indicated by symbols *, and from considerably conserved to relatively conserved.

A 1.5-month-old girl was admitted to our hospital due to persistent diarrhea and failure to thrive. She was the only child of her non-consanguineous parents without any familial history of diarrhea. She had experienced no recurrent infections before. She was born at 39 gestational weeks. Her birth height was 48 cm (10th percentile), and her birth weight was 2.5 kg (below the 3rd percentile) (intrauterine growth retardation). She received mixed feeding (regular formula and breast milk) at birth but was stunted at 1.5 months of age (height, 50 cm, below the 3rd percentile; weight, 3.16 kg, below the 3rd percentile) (Figure 1A). She had non-bloody watery stools seven to eight times a day and poor weight gain since birth. Deep hydrolysis formula and amino-acid formula were administered after diagnosis of allergy to cow-milk protein. However, her condition did not improve.

Physical examination showed that the infant was weak and displayed a severe loss of subcutaneous fat. Her hair was short and sparse (woolly and brittle) (Figure 1B). No abnormalities were observed on the skin. Normal results were shown in all laboratory and imagological examinations.

### Genetic Analysis

WES and Sanger sequencing were performed to identify the causative genes. A pair of novel compound-heterozygous variants, c.3602_3609delAGCGCCTG (p.Q1201Rfs*2) with the maternal origin and c.1990A > G (p.T664A) with the paternal origin, in the *SKIV2L* gene (NM_006,929) were identified via WES and validated via Sanger sequencing (Figure 1C). Variant p. Q1201Rfs*2 can be interpreted as “likely pathogenic” according to the ACMG standard (PVS1_strong + PM2+PP3). This variant is a null mutation which might truncate the protein, but the mutation is located at the last exon (PVS1_strong). The variant is absent from the controls (ExAC v1.0, gnomAD v2.1.1, ESP6500SI-V2, 1kGenomes v3.7.6) (PM2). Multiple lines of *in silico* algorithms, including MutationTaster2 (Schwarz et al., 2014) (1, disease-causing), SIFT Indel (Sim et al., 2012) (0.783, damaging), CADD v1.6 (Rentzsch et al., 2019) (23 > cutoff = 15, deleterious), and CAPIC (Li et al., 2020) (0.941 > cutoff = 0.02, pathogenic), predicted that it is deleterious, except for MutPred-LOF (Pagel et al., 2017) (0.317 < cutoff = 0.6, neutral) (PP3). The other variant c.1990A > G (p.T664A) can be classified as a “variant with uncertain clinical significance” (PM2 + PP3). This missense was not found in the controls (PM2) and also predicted to be deleterious by multiple lines of algorithms (MutationTaster2, 1.000, disease-causing; SIFT v6.2.1 (Sim et al., 2012), 0.00 < cutoff = 0.05; Provean (Choi and Chan, 2015), −4.91 < −2.5, deleterious; and Polyphen-2 (Adzhubei et al., 2013), HumDiv and HumVar, 0.991 and 0.913, probably damaging) (PP3). The missense variant is located in a highly conserved region (Figure 1D), suggesting the importance of the mutated residue. Thus, the changes in residue properties may damage the structure and function of the protein product. We considered both of these variants disease-causing.

### Treatment and Prognosis

The patient was continuously fed with an amino-acid formula through a gastric tube, in addition to parenteral nutrition. Unfortunately, she developed a fever due to an infection from the central venous catheter of the parenteral nutriton. The infection was controlled by administrating cefoperazone sodium and sulbactam sodium. Additionally, as the volume of the fed amino-acid formula was increased, the amount of the intravenous nutrient fluid was gradually decreased until the parenteral nutrition was finally discontinued. Finally, she was discharged at 3 months of age and thereafter fed with 20 ml of an amino-acid formula per hour through a gastric tube. The recent follow-up at the age of 7.5 months revealed that the child still grew slowly (height, 61 cm, below the 3rd percentile; weight, 4.25 kg, below the 3rd percentile) and had intermittent diarrhea.

## Discussion and Conclusion

THES2 is a rare and severe genetic disorder known to be associated with pathogenic variants of *SKIV2L*. Based on previous reports (Fabre et al., 2012; Fabre et al., 2013; Morgan et al., 2013; Monies et al., 2015; Lee et al., 2016; Zheng et al., 2016; Bick et al., 2017; Hiejima et al., 2017; Bourgeois et al., 2018; Vardi et al., 2018; Rudilla et al., 2019; Fung et al., 2020; Taher et al., 2020; Dyment et al., 2021; Klee et al., 2021), the incidence of this disease is not significantly different between genders (M:F = 17:20) (Table 1). Patients have their onset mostly in the neonatal period, ranging from birth to 0.8 years of age (mean, 29.5 days; median, 17 days). The symptoms of the current patient were noticed at birth. All THES2 patients have intractable diarrhea (34/34, 100%). Moreover, THES2 is also associated with intrauterine growth restriction (25/28, 89.3%), hair abnormalities (31/35, 88.6%), facial dysmorphisms (24/31, 77.4%), hepatic involvement (19/27, 70.4%), immunodeficiency (16/30, 53.3%), and cardiac abnormalities (7/19, 36.8%). This disease has been described in Middle-Eastern (11/37, 29.7%), European (10/37, 27.0%), Asian (7/37, 18.9%), American (5/37, 13.5%), and African (4/37, 10.8%) populations, with the majority seen in the Middle-Eastern background.

**TABLE 1:** Genotypic and phenotypic features of THES2 and the corresponding *SKIV2L* mutations.

References	Mutations in *SKIV2L* gene	Background	GENDER (M:F = 17:20)	Onset age	Intrauterine growth restriction (25/28)	Intractable diarrhea (34/34)	Hair abnormalities (31/35)	Facial dysmorphisms (24/31)	Hepatic involvement (19/27)	Immunodeficiency (16/30)	Cardiac abnormalities (7/19)	Deceased (3/19)	Other features
PRESENT CASE	c.1990A > G (p.T664A) and c.3602_3609delAGCGCCTG (p.Q1201Rfs*2)	Asian	F	At birth	+	+	+	−	−	v	−	−	Physical developmental delay
Fabre et al. (2012)	c.848G > A (p.W283*) and c.1022T > G (p.V341G)	European	F	1–12 weeks (median 2.5 and mean 3.8 weeks)	+ (4/6)	+ (6/6)	+ (6/6)	+ (6/6)	+ (3/6)	+ (3/6)	+ (2/4)	+ (2/4)	Villous atrophy (3/5), colitis (3/3), siderosis (1/3), cirrhosis (2/3)
	c.1434delT (p.S479Afs*3)	Middle-Eastern	F										
	c.1635_1636insA (p.G546Rfs*35)	African	F										
	c.2266C > T (p.R756*) and c.2442G > A (p.W814*)	European	F										
	c.2572delG (p.V858*)	Middle-Eastern	M										
	c.2662_2663delAG (p.R888Gfs*12)	Middle-Eastern	F										
Fabre et al. (2013)	c.3561_3581delGCTCTCAGGGACCCCTGAGGG (p.S1189_L1195del)	European	M	NA	+	+	+	+^5^	NA	+	NA	−	−
Bourgeois et al. (2018)													
Morgan et al. (2013)	c.3391delC (p.L1131Sfs*5)	Asian	F	2 weeks	+	+	+	NA	+	+	NA	+ (9 months)	Villous atrophy, platelet abnormalities
Monies et al. (2015) Bourgeois et al. (2018)	c.3561_3581del GCT​CTC​AGG​GAC​CCC​TGA​GGG (p.S1189 _L1195del)^a^	Middle-Eastern	F	10 days	NA	+	+	+	+	−	NA	−	Failure to thrive, peg teeth, hyperpigmentation, mental retardation
			M	14 days	NA	+	+	+	+	−	NA	−	Failure to thrive, hyperpigmentation
			F	14 days		+	+	+	NA	−		−	Failure to thrive, peg teeth, hyperpigmentation
			M	4 days		+	−	+	NA	−		−	Failure to thrive, hyperpigmentation
Lee et al. (2016)	c.1891G > A (p.G631S) and c.3187C > T (p.R1063*)	Asian	M	17 days	+	+	−	+	−	−	+	−	Jejunal villous atrophy, thrombocytosis, developmental delay, poor dentition
			M	1 month	+	+	−	+	+	NA	+	−	Thrombocytosis
Zheng et al. (2016)	c.1120C > T (p.R374*) and c.1891G > A (p.G631S)	Asian	M	4 weeks	+	+	+	+	+	+	NA	−	Failure to thrive
Hiejima et al. (2017)	c.1420C > T (p.Q474*) and c.3262G > T (p.E1088*)	Asian	F	14 days	+	+	+	+	+	−	−	−	Mild mental retardation, failure to thrive, villous atrophy
Bick et al. (2017)	c.2203-1G > C and c.3187C > T (p.R1063*)	American	F	NA	+	+	+	NA	+	+	+	NA	Developmental delay, hearing abnormalities, hypothyroidism, protein losing enteropathy
Vardi et al. (2018)	c.1891G > A (p.G631S)	Middle-Eastern	F	10 days	+	+	+	−	−	+	NA	−	Sepsis, electrolyte imbalance, convulsions, anemia, respiratory distress
Bourgeois et al. (2018)	c.919-1G > A and c.2341–2A > G	European	F	NA	+	+	+	+	+	+	−	NA	Skin problem
	c.994G > C (p.A332P) and c.3103_3105delAAG (p.K1035del)^b^	European	M		-	+	+	−	+	+	−		−
	c.1312G > A (p.E438K)	African	M		NA	+	+	+	+	−	−		Skin problem
	c.1396T > G (p.W466G)	African	M		+	+	+	-	+	−	−		−
	c.1434delT (p.S479Afs*3)	Middle-Eastern	F		+	+	+	+	NA	NA	NA		−
	c.1447C > T (p.R483C)	African	M		+	+	−	−	+	+	−		−
	c.1452delC (p.V485Cfs*45)^c^ and c.2498_2499delTG (p.V833Efs*45)	European	M		+	+	+	+	+	−	−		Skin problem
	c.1528C > T (p.R510*)	American	F		NA								Monoallelic mutation
	c.1528C > T (p.R510*)	American	F		NA								Monoallelic mutation
	c.3113_3141del (p.E1038Vfs*7)^d^	European	M		+	+	+	+	+	+	+		-
	c.3172C > T (p.R1058*)	Middle-Eastern	M		+	+	+	+	+	+	−		Skin problem
	c.3187C > T (p.R1063*)	European	F		+	+	+	+	+	+	+		-
	c.3187C > T (p.R1063*)	European	F		+	+	+	NA	NA	NA	NA		Monoallelic mutation
	c.3561_3581delGCTCTCAGGGACCCCTGAGGG (p.S1189_L1195del)	NA	M		NA	NA	+	+	NA	NA	NA		−
	c.3561_3581delGCTCTCAGGGACCCCTGAGGG (p.S1189_L1195del)	NA	M		+	+	+	NA	NA	NA	NA		−
Rudilla et al. (2019)	c.2203-1G > A and c.3187C > T (p.R1063*)	European	M	0.8 years	NA	+	+	−	−	+	−	−	Short stature, psychomotor delay
Fung et al. (2020)	c.1404–2A > G and c.1647+1G > A	Asian	NA										
Taher et al. (2020)	c.1297C > T (p.R433C)	Middle-Eastern	F	8 days	+	+	+	−	−	−	NA	−	Global developmental delay, bilateral inguinal hernia, failure to thrive
Dyment et al. (2021)	c.235C > T (p.R79*)^f^	American	F	NA	+	+	+	+	NA	+	NA	−	Failure to thrive, psychomotor development delay, skin and hearing abnormalities, strabismus, sacral dimple, autism
Klee et al. (2021)	c.1211G > A (p.R404H)	American	NA										

Note: F, female; M, male; THES2, Trichohepatoenteric Syndrome 2; *SKIV2L*, superkiller viralicidic activity 2-like; NA, Not available.

a Previously described as c.3559_3579del (p.1187_1193del) in Monies et al., 2015 [39].

b Previously described as c.3101_3,103 delAGA (p.Gln1034del) in Bourgeois et al., 2018 [7].

c Previously described as c.1452delC (p.Pro484fs*46) in Bourgeois et al., 2018 [7].

d Previously described as c.3112_3,140 del (p.Glu1038fs*7) in Bourgeois et al., 2018 [7].

e We considered “dysmorphy” in Bourgeois et al., 2018 [7] as facial dysmorphisms here.

f The patient was diagnosed with Dubowitz syndrome in Dyment et al., 2021 [45].

According to the HGMD v2021.8, only 35 mutations in *SKIV2L*, including 32 disease-causing mutations (DM) and 3 possibly disease-causing mutations (DM?), have been reported to be associated with THES2. Nonsense mutations (10/35, 28.6%) are the most prevalent type of mutations, followed by missense (8/35, 22.9%), frameshift (7/35, 20.0%), canonical splice site (7/35, 20.0%), large-size (>20 bp) deletion (2/35, 5.7%), and inframe deletion (1/35, 2.9%) mutations. Although THES2 is considered an autosomal recessive disorder, three patients have been reported to carry a heterozygous *SKIV2L* mutation (Bourgeois et al., 2018). In-depth genetic analyses focusing on deep-intronic mutations may explain this discrepancy.

THES2 is usually diagnosed based on both the common THES symptoms and biallelic pathogenic variants of the *SKIV2L* gene. However, no phenotype–genotype correlation has been established to date. How *SKIV2L* mutations or defects in the Ski complex lead to the observed symptoms is still unclear. It has been revealed that SKIV2L RNA exosome limits the activation of RIG-I-like receptors (RLR), thereby negatively regulating RLR-mediated antiviral response. Human patients with SKIV2L mutations also have a potent type I interferon signature in the blood cells (Eckard et al., 2014). Such uncontrolled regulations and overwhelming responses could probably drive immune disease, leading to chronic intestinal inflammation, i.e., intractable diarrhea. Given that SKI2W is a helicase with ATPase activity, SKI2W defects may affect the normal function of the Ski complex, and consequently, the mRNA targets of this mRNA surveillance are not degraded, whereby the observed phenotypes ensue. Additional research is required to determine the pathogenesis of THES2 and the relationships between mutations and the symptoms.

Most patients with THES may need lifelong total parenteral nutrition (TPN) to survive, but some of them develop complications associated with TPN. To date, only a few infants with THES have attained normal oral nutrition (Fabre et al., 2017). In the current study, the patient was fed with an amino-acid formula and parenteral nutrition but subsequently developed complications similar to those associated with TPN. It seems that a small supplement of amino acids can be therapeutic for THES2 patients, but extreme cases might require extraoral administration of an extensively hydrolyzed formula (Taher et al., 2020). THES2 is a severe and potentially fatal disorder; however, only a few patients have been reported to die from THES2 (Fabre et al., 2012; Taher et al., 2020). Nevertheless, most cases lacked long-term follow-up. Therefore, a long follow-up period is required for patients with THES2 to acquire information on the prognosis and effectiveness of the clinical interventions.

In summary, this study reported a pair of novel compound-heterozygous variants of the *SKIV2L* gene in a patient with THES2, thereby expanding the spectrum of the causative mutations. Early genetic diagnosis of this syndrome could enable early and proper treatment. An amino-acid formula continuously fed through a gastric tube, alongside parenteral nutrition, could also delay the complications and improve the outcome of such cases.

## Data Availability

The datasets for this article are not publicly available due to concerns regarding participant/patient anonymity. Requests to access the datasets should be directed to the corresponding author.
